# Plasma GAS6 predicts mortality risk in acute heart failure patients: insights from the DRAGON-HF trial

**DOI:** 10.1186/s12967-022-03859-w

**Published:** 2023-01-12

**Authors:** Teng Ma, Rongrong Huang, Yanhua Xu, Yangbo Lv, Yifan Liu, Xin Pan, Jia Dong, Di Gao, Zeyu Wang, Fenglei Zhang, Chunxi Yan, Sang-Bing Ong, Yang Su, Dachun Xu

**Affiliations:** 1 Department of Cardiology, Shanghai Tenth People’s Hospital, Tongji University School of Medicine, 301 Middle Yanchang Road, Shanghai, 200072 China; 2 Department of Cardiology, Qidong People’s Hospital, Qidong, Jiangsu China; 3 Department of Cardiology, Shanghai Yoda Cardiothoracic Hospital, Shanghai, 200012 People’s Republic of China; 4 Department of Geriatrics, Shanghai Tenth People’s Hospital, Tongji University, Shanghai, China; 5 Department of Cardiovascular, Affiliated Hospital of Shaanxi University of Traditional Chinese Medicine, Xianyang, China; 6 Department of Cardiology, Shanghai Xuhui District Central Hospital & Zhongshan-Xuhui Hospital, Shanghai, China; 7 Department of Medicine and Therapeutics, Faculty of Medicine, Chinese University of Hong Kong (CUHK), Hong Kong, SAR China; 8 Centre for Cardiovascular Genomics and Medicine (CCGM), Lui Che Woo Institute of Innovative Medicine, CUHK, Hong Kong, SAR China

**Keywords:** Growth arrest-specific 6, Acute heart failure, Prognosis, Cohort study, Biomarkers

## Abstract

**Background:**

Growth arrest-specific 6 (GAS6) is a vitamin K-dependent protein related to inflammation, fibrosis, as well as platelet function. Genetic ablation of GAS6 in mice protects against cardiac hypertrophy and dysfunction. Nonetheless, the association between plasma GAS6 levels and acute heart failure (AHF) patients is still unknown.

**Methods:**

We measured plasma GAS6 concentrations in 1039 patients with AHF who were enrolled in the DRAGON-HF trial (NCT03727828). Mean follow-up of the study was 889 days. The primary endpoint is all-cause death.

**Results:**

In total, there were 195 primary endpoints of all-cause death and 135 secondary endpoints of cardiovascular death during the mean follow-up duration of 889 days. The higher levels of GAS6 were associated with higher rates of all-cause and cardiovascular death (P < 0.05). Baseline plasma GAS6 levels were still strongly correlated with clinical outcomes in different models after adjustment for clinical factors and N-terminal pro-brain natriuretic peptide (NT-proBNP, P < 0.05). GAS6 could further distinguish the risks of clinical outcomes based on NT-proBNP measurement.

**Conclusion:**

Elevated plasma GAS6 levels were associated with an increased risk of all-cause and cardiovascular death in patients with AHF.

*Trial registration* NCT03727828 (DRAGON-HF trial) *clinicaltrials.gov*

**Supplementary Information:**

The online version contains supplementary material available at 10.1186/s12967-022-03859-w.

## Background

Heart failure (HF) is a devastating disease that remains a leading cause of death worldwide and is a primary socioeconomic burden for many countries. The mortality rate from acute heart failure (AHF) remains high despite current progress in HF treatment. As such, improved risk stratification and prognostic analysis tools are required to help guide the management of AHF patients. To achieve enhanced accuracy while not compromising convenience, novel biomarkers in plasma may enhance diagnostic and prognostic values in HF [1, 2].

In this regard, Growth arrest-specific 6 (GAS6) is a vitamin K-dependent protein which has been detected in many different tissues and cells [3–5]. Biological functions of GAS6 are regulated by interaction with the tyrosine kinase receptor family (TAM): TYRO3 Protein Tyrosine Kinase (Tyro3), AXL, MER Proto-Oncogene, Tyrosine Kinase (MERTK), with the highest affinity for AXL. The extracellular domain of these receptors can be proteolytically cleaved and released into the plasma in soluble forms to act as decoy receptors of GAS6, which reduces interaction between the ligand and receptor [6, 7]. GAS6/TAM interaction has been implicated in platelet function regulation [8], cell growth [9], phagocytosis of apoptotic cells [10], and reduction of inflammatory response [11]. GAS6 has been regarded as a risk factor for adverse effects in many diseases, such as systemic lupus erythematosus [12, 13], rheumatoid arthritis [14], liver cirrhosis [15] and venous thromboembolism [16, 17]. Although GAS6 showed relatively low expression levels in heart tissue under a normal situation, it has been demonstrated to increase significantly in hypertrophic and failing hearts [18]. Our previous study had demonstrated that *Gas6* knockout would alleviate cardiac hypertrophy, fibrosis and contractile dysfunction [18]. A previous study also found GAS6 levels to be significantly increased at day 7 post-admission in ST elevation myocardial infarction (STEMI) patients [19]. Nonetheless, the association between GAS6 and clinical prognosis in patients with AHF remains unclear. In this study, we investigated the prognostic value of GAS6 in a cohort of AHF patients which enrolled in the DRAGON-HF trial.

## Methods

### Study population

The study enrolled 1039 patients between January 2017 and March 2020 with age > 18 yrs, symptomatic (NYHA class II-IV) heart failure. The diagnostic criteria of AHF referred to the 2016 ESC guidelines [20]. Patients with AHF was diagnosed by two clinicians based on a thorough history assessing symptoms, prior cardiovascular history and potential cardiac and non-cardiac precipitants. Assessment of signs /symptoms of congestion and /or hypoperfusion by physical examination and further confirmed by appropriate additional investigations such as ECG, chest X-ray, laboratory assessment (with specific biomarkers) and echocardiography. Patients with BNP < 100 pg/mL and/or NT-proBNP < 300 pg/mL were excluded for the diagnosis of AHF (based on sex, age, BMI and renal function). Patients with serious infectious diseases, severe hepatic and renal dysfunction, tumor and blood diseases were excluded from our study. In our study, we also identified AHF patients with renal dysfunction as evaluated by eGFR < 60 mL/min/1.73m^2^, which constitutes a part of the observational, cohort trial: DRAGON-HF trial (Diagnostic, Risk Stratification and Prognostic Value of Novel Biomarkers in Patients with Heart Failure, NCT03727828). This study complied with the Declaration of Helsinki and was approved by the local ethics committee. Informed consent was obtained from all patients. Patients and the public were not involved in the design, or conduct, or reporting, or dissemination plans of our study.

### Plasma sampling

Following provision of consent and with the patient recumbent for at least 15 min, 20 mL of venous blood was withdrawn by venipuncture of an antecubital vein and collected in evacuated tubes containing EDTA as anticoagulant. Blood samples were centrifuged at 1500*g* for 20 min at 4 ℃. Plasma was siphoned, aliquoted and shipped on dry ice to a central laboratory. Samples were stored at − 80 ℃ until analysis. At the time of assaying, plasma samples were defrosted at room temperature and analyzed in a single batch.

### Biomarkers assessment

Circulating plasma concentration of GAS6 was measured by a commercially available ELISA assay (catalog: DY885B) as previously described [21]. According to the manufacturer, the limit of detection of this assay is 15.6 pg/mL and the upper reference limit is 1000 pg/mL. The intra-assay and inter-assay coefficients of variation were 6.5% and 8.5%, respectively. NT-proBNP levels were determined by the clinically available VITROS® NT-proBNP Assay (Ortho-Clinical Diagnostics, Raritan, NJ, USA).

### Endpoints

The primary endpoint of the study is all-cause death. The secondary endpoint is cardiovascular death.

### Statistical analysis

Continuous variables were presented as mean ± standard deviations or median (IQR). One way ANOVA test or Mann–Whitney U test was used to examine the difference between groups depending on the data distribution. Categorical variables were assessed by Chi-Squared test in different groups. The correlation between GAS6 and eGFR was visualized with scatterplots, and the Spearman correlation coefficient was calculated. Kaplan–Meier method was used to derive the cumulative endpoint-free survival estimates. Survival curves stratified according to the tertile distribution of GAS6 were compared with the log-rank test for trend. We also tested the association between GAS6 and long-term outcomes in different clinical models. Ln-transformed biomarker distributions were standardized to a mean of 0 and standard deviation of 1 to facilitate comparison of effect sizes between biomarkers. We first evaluated the GAS6 in models containing the following standard clinical risk factors: age, sex, regular cigarette smoking, body mass index, eGFR, presence of diabetes mellitus, hypertension, and atrial fibrillation, medical usage of diuretics and spironolactone. We then performed analyses incorporating all biomarkers together and add NT-proBNP, hs-TnT and hs-CRP to the clinical model. We also assessed the improvement of prediction value by IDI and NRI. The corresponding C-index, reflecting the discriminative ability of each biomarker up to mediate follow-up 889 days, was calculated. Proportional hazards assumption was confirmed by Schoenfeld’s test. All data were analyzed using IBM SPSS Statistics (version 20, 2011) and R statistical software (http://www.r-project.org/, version 4.1.0). The C-index, NRI, and IDI were calculated in R software, packages SurvC1 and Survival. Two-sided P-values < 0.05 were considered statistically significant.

## Results

### Patients characteristics

Baseline characteristics are shown in Table 1. Among 1039 patients recruited, 669 (64.4%) were men and median age was 71 yrs (IQR 64–81). Median follow-up time was 889 days. Mean plasma concentration of GAS6 in these patients was 10.34 ng/mL. Patients with higher GAS6 levels were older and more likely to exhibit lower left ventricular ejection fraction (LVEF), more severe HF (evaluated by NYHA functional class) and reduced renal function (assessed by eGFR). Furthermore, blood urea nitrogen and creatinine are directly proportionate to GAS6 levels. Meanwhile, a higher level of GAS6 in patients of AHF increases the prevalence of hypertension, alongside an incremental proportion of β-blocker, spironolactone and diuretic usage. Patients with high GAS6 levels also had enhanced hs-TnT, hs-C-reactive protein (hs-CRP) and decreased eGFR, although GAS6 levels were modestly correlated with hs-TnT (r = 0.196, P < 0.001), hs-CRP (r = 0.214, P < 0.001) and eGFR (r = − 0.137, p < 0.001). In contrast, GAS6 levels showed no correlation to body mass index, systolic blood pressure, heart rates, hemoglobin, smoking history, diabetes mellitus, atrial fibrillation, ACEI/ARBs, calcium channel blockers and statins.

**Table 1:** Baseline characteristics of patients

Clinical characteristics	All (1039)	GAS6			P value
		< 8.6 ng/mL (344)	8.6–12.0 ng/mL (349)	≥ 12.0 ng/mL (346)	
Age, years	71 (64–81)	70 (64–78)	71 (63–81)	74 (64–82)	0.02
Sex, male, n (%)	669 (64.4)	210 (61.0)	222 (63.6)	237 (68.5)	0.12
BMI, kg/m^2^	24.4 ± 3.6	24.7 ± 3.5	24.1 ± 3.6	24.3 ± 3.7	0.14
HR, beats/minute	81 ± 18	80 ± 17	81 ± 18	83 ± 18	0.12
SBP, mm/Hg	136 ± 23	137 ± 22	135 ± 23	136 ± 25	0.40
DBP, mm/Hg	77 ± 15	78 ± 14	76 ± 15	77 ± 16	0.27
NYHA class					0.001
II	674 (65.7)	247 (72.9)	226 (65.5)	201 (58.8)	
III	290 (28.3)	80 (23.6)	99 (28.7)	111 (32.5)	
IV	62 (6.0)	12 (3.5)	20 (5.8)	30 (8.8)	
Echocardiography					
LVEF, %	47 ± 13	49 ± 13	47 ± 14	46 ± 13	0.004
LVDD, mm	50 ± 9	49 ± 8	51 ± 9	51 ± 9	0.04
LVDS, mm	36 ± 11	35 ± 10	37 ± 11	37 ± 11	0.002
IVS, mm	11 ± 4	10 ± 2	11 ± 5	11 ± 4	0.96
LAS, mm	42 ± 7	42 ± 7	43 ± 8	43 ± 7	0.04
LVPWD, mm	10 ± 2	10 ± 1	10 ± 1	10 ± 3	0.67
LVMI, mm	115 ± 37	109 ± 35	119 ± 38	116 ± 37	< 0.001
Medical history					
Smoking, n (%)	236 (22.7)	78 (22.7)	74 (21.2)	84 (24.3)	0.30
Hypertension, n (%)	698 (67.2)	237 (68.9)	215 (61.6)	246 (71.1)	0.02
Diabetes mellitus, n (%)	352 (33.9)	109 (31.7)	117 (33.5)	126 (36.4)	0.42
Atrial fibrillation, n (%)	289 (27.8)	94 (27.3)	101 (28.9)	94 (27.2)	0.85
Chronic obstructive pulmonary disease, n (%)	35 (3.4)	7 (2.0)	10 (2.9)	18 (5.2)	0.06
Myocardial infarction, n (%)	245 (23.6)	73 (21.2)	78 (22.3)	94 (27.2)	0.15
Drug usage					
ACEI, n (%)	138 (13.3)	45 (13.1)	44 (12.6)	49 (14.2)	0.83
ARB, n (%)	444 (42.7)	164 (47.7)	142 (40.7)	138 (39.9)	0.08
β-blocker, n (%)	766 (73.7)	237 (68.9)	262 (75.1)	267 (77.2)	0.04
CCB, n (%)	280 (26.9)	100 (29.1)	81 (23.2)	99 (28.6)	0.15
Spironolactone, n (%)	477 (45.9)	130 (37.8)	169 (48.4)	178 (51.4)	0.001
Diuretics, n (%)	520 (50.0)	140 (40.7)	181 (51.9)	199 (57.5)	< 0.001
Statins, n (%)	841 (80.9)	286 (83.1)	282 (80.8)	273 (78.9)	0.37
Antiplatelets, n (%)	856 (82.4)	284 (82.6)	289 (82.8)	283 (81.8)	0.94
Anticoagulants, n (%)	502 (48.3)	145 (42.2)	181 (51.9)	176 (50.9)	0.02
Laboratory results					
HB, Hg/L	128 ± 19	130 ± 19	128 ± 18	127 ± 21	0.15
eGFR, mL/min/1.73 m^2^	78 ± 30	81 ± 30	80 ± 29	72 ± 31	< 0.001
BUN, mmol/L	6.7 (5.3–8.8)	6.1 (5.1–8.0)	6.7 (5.2–8.9)	7.1 (5.7–9.7)	< 0.001
CB, μmol/L	3.3 (2.7–5.2)	3.2 (2.5–4.9)	3.3 (2.8–5.3)	3.2 (3.0–5.5)	0.18
UCB, μmol/L	7.8 (5.2–12.1)	7.5 (5.1–11.2)	7.9 (5.2–12.2)	8.3 (5.3–12.9)	0.27
Cr, μmol/L	87 (73–111)	86 (72–105)	84 (71–107)	93 (75–125)	< 0.001
TnT, ng/mL	0.029 (0.015–0.088)	0.022 (0.013–0.057)	0.029 (0.015–0.080)	0.036 (0.018–0.158)	< 0.001
CRP, mg/L	3.5 (3.0–12.2)	3.2 (3.0–7.2)	3.2 (3.0–9.8)	5.4 (3.0–19.5)	< 0.001
NT-proBNP, pg/mL	1460 (661–3764)	853 (464–2290)	1563 (822–4428)	2171 (968–5519)	< 0.001

BMI: body mass index; HR: heart rate; SBP: systolic blood pressure; DBP: diastolic blood pressure; NYHA: New York Heart Association; LVEF: left ventricular ejection fraction; LVDD: left ventricular diastolic diameter; LVDS: left ventricular systolic diameter; IVS: interventricular septum; LAS: left atrium end systolic diameter; LVPWD: left ventricular posterior wall thickness; LVMI: left ventricular mass index; ACEI: angiotensin-converting enzyme inhibitors; ARB: angiotensin receptor blocker; CCB: calcium channel blockers; Hb: hemoglobin, eGFR: estimate glomerular filtration rate; BUN: blood urea nitrogen; Cr: creatinine; CB: conjugated bilirubin; UCB: unconjugated bilirubin; TnT: troponin T; CRP: C-reactive protein; NT-proBNP: N-terminal pro-B-type natriuretic peptide

### Baseline GAS6 levels and clinical outcomes

**Fig. 1:**
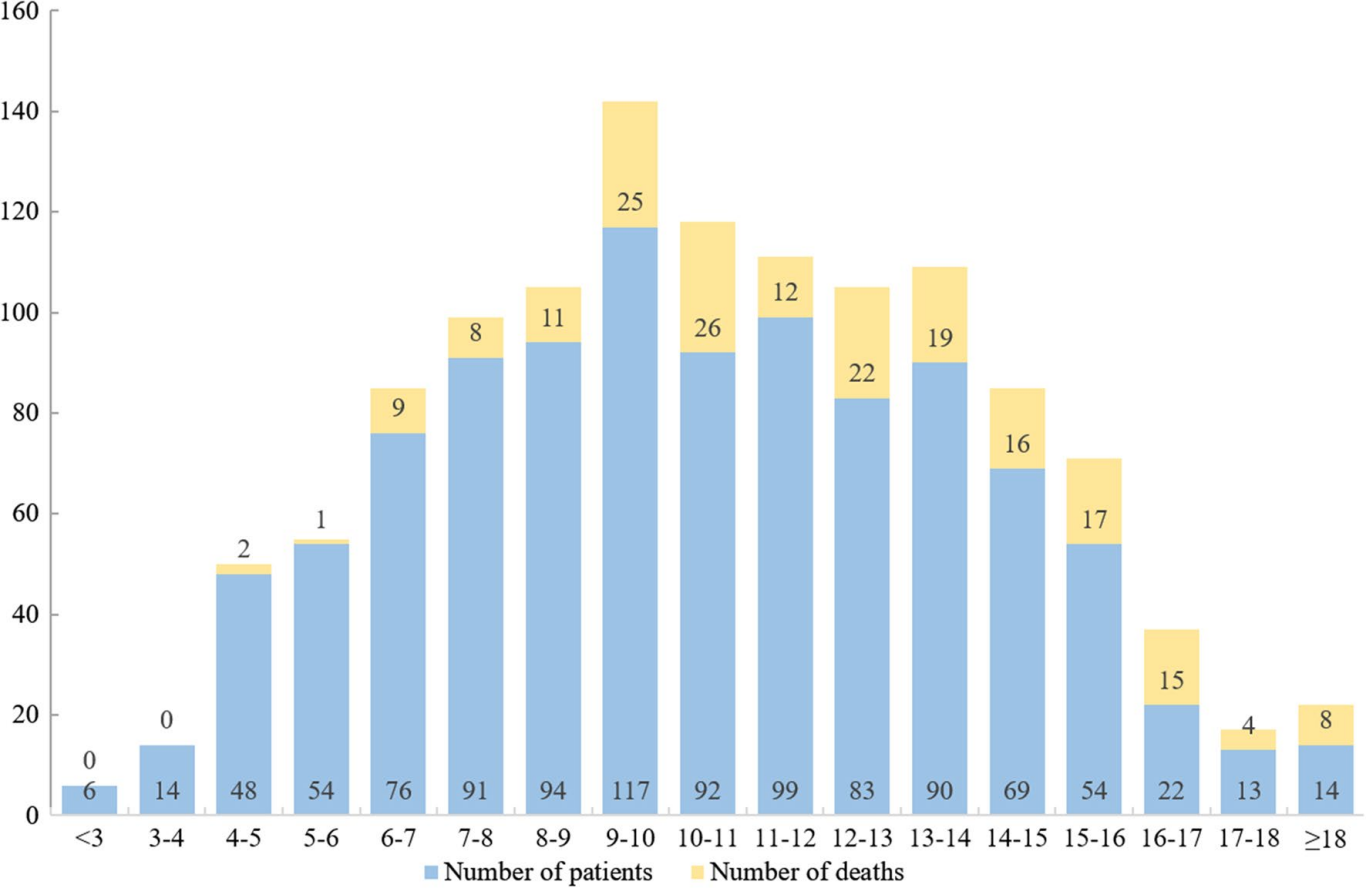
Distribution of the number of patients and deaths according to GAS6 levels

**Fig. 2:**
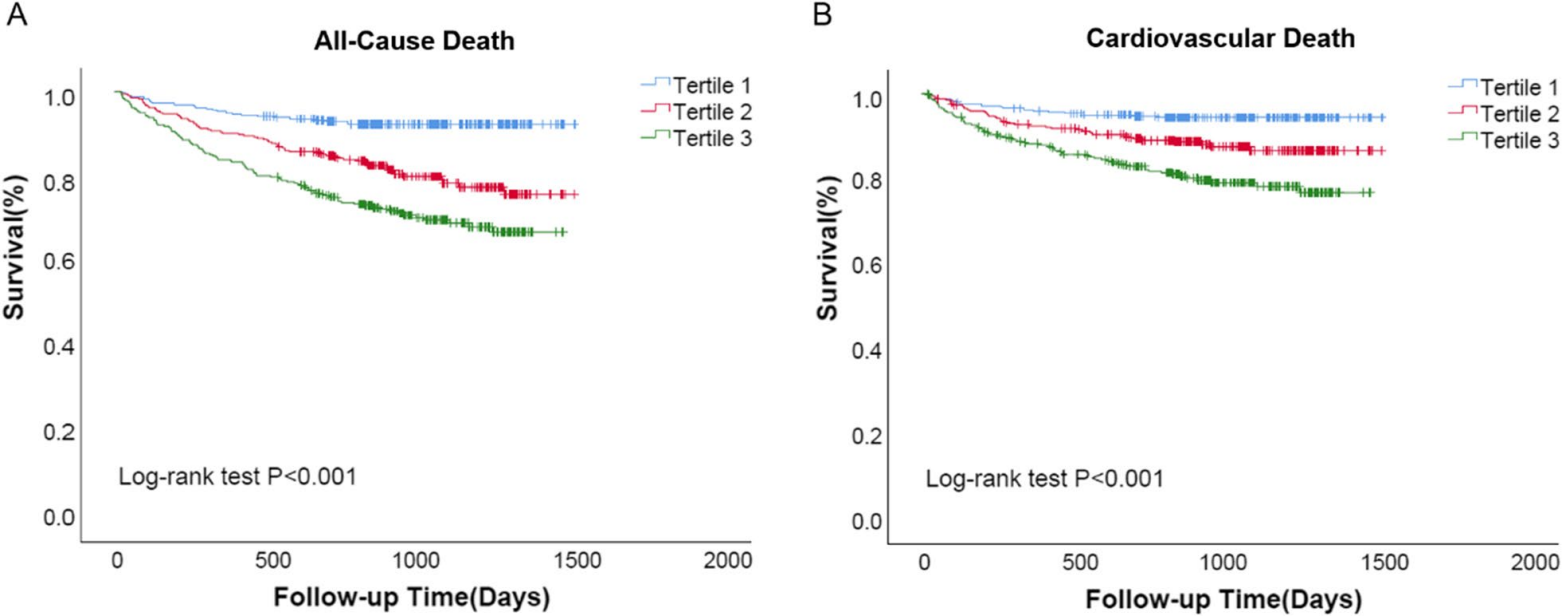
Kaplan–Meier curves for all-cause death and cardiovascular death according to GAS6 levels

During a median follow-up of 889 days (interquartile range 728–1182 days), 195 (18.8%) patients died from all-causes and 128 died from cardiovascular causes. Endpoint-free survival stratified according to the distribution of GAS6 levels indicated that patients with lower GAS6 levels (Tertile 1; GAS6 < 8.60 ng/mL) had a significantly better clinical endpoint-free survival than patients with higher GAS6 levels (GAS6 ≥ 12.0 ng/mL) (as shown in Figs. 1 and 2). However, there’s no significant difference in HF re-hospitalization among tertiles (data not shown).

Univariable and multivariable analysis with categorical and continuous GAS6 levels and adjustment models presented a significant relation between GAS6 and clinical endpoints (Table 2 and Additional file 1: Table S1). In model 1, GAS6 levels were associated with increasing all-cause death (HR, 1.65; 95% CI (1.41,1.93); P < 0.001) and cardiovascular death (HR, 1.71; 95% CI (1.40,2.09); P < 0.001). After additionally adjusting for clinical covariates, GAS6 levels remained associated with all-cause death (HR, 1.56; 95% CI (1.33,1.83); P < 0.001) and cardiovascular death (HR, 1.60; 95% CI (1.30,1.95); P < 0.001). After adding biomarkers into traditional clinical model, which includes age, sex, BMI, smoking, hypertension, diabetes, atrial fibrillation and eGFR, GAS6 levels were also significantly associated with all-cause death (HR, 1.44; 95% CI (1.22,1.70); P < 0.001) and cardiovascular death (HR, 1.44; 95% CI (1.17,1.79); P < 0.001).

**Table 2:** Associations between GAS6 and all-cause death or cardiovascular death, with adjustment for clinical characteristic

	95% CI	P value	c-index
All-cause death			
Model 1	1.65 (1.41,1.93)	< 0.001	0.70 (0.66,0.74)
Model 2	1.56 (1.33,1.83)	< 0.001	0.79 (0.76,0.83)
Model 3	1.44 (1.22,1.70)	< 0.001	0.81 (0.78,0.85)
Cardiovascular death			
Model 1	1.71 (1.40,2.09)	< 0.001	0.68 (0.63,0.73)
Model 2	1.60 (1.30,1.95)	< 0.001	0.81 (0.77,0.85)
Model 3	1.44 (1.17,1.79)	0.001	0.83 (0.80,0.87)

Standardized hazard ratios, reflecting the instantaneous risk of the primary and secondary endpoint per one standard deviation increase in the ln-transformed biomarker level

Model 1: age, sex, GAS6;

Model 2: age, sex, BMI, smoking, hypertension, diabetes, atrial fibrillation, eGFR, diuretics, Spironolactone, GAS6;

Model 3: age, sex, BMI, smoking, hypertension, diabetes, atrial fibrillation, eGFR, diuretics, spironolactone, GAS6, NT-proBNP, TnT, CRP

### Biomarkers for prediction of all-cause death and cardiovascular death

Univariate and multivariate Cox proportional hazards analysis for clinical outcomes showed several variables were independent predictors of all-cause death and cardiovascular death in patients with AHF. For comparing the effect size of GAS6 and classic biomarkers in predicting the adverse outcome, we selected several variables as clinical risk factors and added biomarkers to the clinical model respectively. Separate incorporation of each biomarker into the clinical risk model showed that all biomarkers increased the C statistic for prediction of adverse events except CRP (C statistics for all-cause death: clinical risk alone 0.74, clinical risk + GAS6 0.77, clinical risk + NT-proBNP 0.78, clinical risk + hs-CRP 0.74 and clinical risk + hs-TNT 0.76. C statistics for cardiovascular death: clinical risk alone 0.74, clinical risk + GAS6 0.77, clinical risk + NT-proBNP 0.80, clinical risk + hs-CRP 0.75 and clinical risk + hs-TNT 0.77) (Table 3) Moreover, we examined the additive usefulness of GAS6 in multiple biomarkers strategy based on clinical risk factors and other biomarkers (C statistics for all-cause death: 0.80 and for cardiovascular death: 0.82). NRI and IDI showed the similar improvement of prediction ability by adding each or all biomarker to the clinical risk model.

**Table 3:** Associations between biomarkers and prognostic discrimination

Model	C-index	NRI	P value	IDI	P value
All-cause death					
Clinical model	0.74	Reference		Reference	
+ GAS6	0.77	0.04 (0.02,0.07)	< 0.001	0.05 (0.01,0.23)	0.03
+ NT-proBNP	0.78	0.08 (0.04,0.11)	< 0.001	0.37 (0.24,0.43)	< 0.001
+ CRP	0.74	0 (0,0.01)	0.909	0.1 (0.12,0.19)	0.37
+ TnT	0.76	0.03 (0.01,0.06)	0.01	0.24 (0.11,0.34)	0.01
NT-proBNP + GAS6 + TnT + CRP	0.80	0.11 (0.07,0.16)	< 0.001	0.35 (0.21,0.45)	< 0.001
Cardiovascular death					
Clinical model	0.74	Reference		Reference	
+ GAS6	0.77	0.04 (0.01,0.08)	< 0.001	0.07 (0,0.26)	0.046
+ NT-proBNP	0.80	0.08 (0.04,0.12)	< 0.001	0.34 (0.25,0.46)	< 0.001
+ CRP	0.75	0 (0,0.02)	0.9	0.11 (0.14,0.22)	0.44
+ TnT	0.77	0.02 (0,0.07)	0.01	0.24 (0.09,0.36)	0.01
NT-proBNP + TnT + CRP + GAS6	0.82	0.11 (0.07,0.18)	< 0.001	0.38 (0.26,0.49)	< 0.001

IDI: integrated discrimination improvement; NRI: net reclassification index; other abbreviations as shown in Table 1

Clinical model includes: age, sex, BMI, smoking, hypertension, diabetes, atrial fibrillation, eGFR

**Fig. 3:**
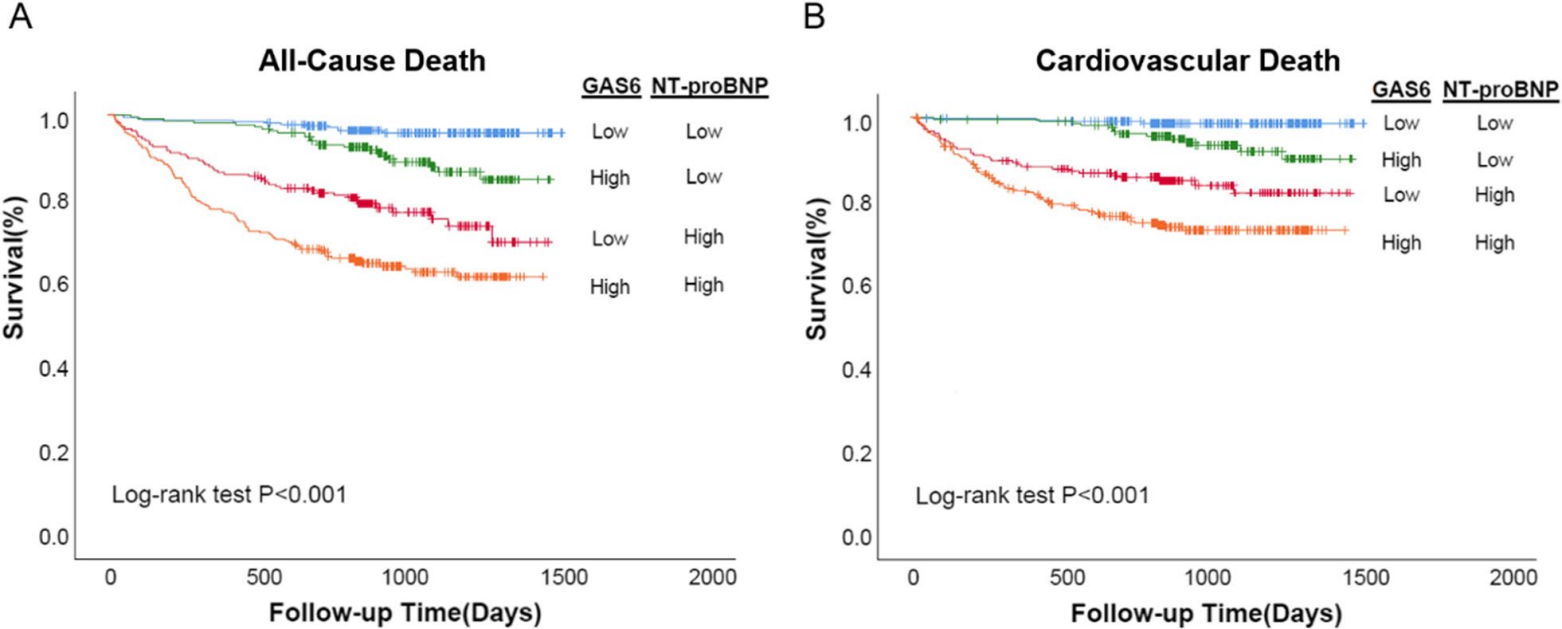
Kaplan–Meier curves for all-cause death and cardiovascular death according to GAS6 and NT-proBNP levels. Baseline median GAS6 was 10.15 ng/mL; the median level for NT-proBNP was 1460.5 pg/mL. ‘High’ refers to values above the median, while ‘low’ refers to values below the median

Apart for the information provided by NT-proBNP, GAS6 provides beneficial prognostic information of AHF patients. Among the patients with baseline NT-proBNP less than the cohort median (< 1460.5 pg/mL), all-cause death was significantly different between the high (> 10.15 ng/mL) and low (< 10.15 ng/mL) GAS6 levels patients (as shown in Fig. 3; log-rank P < 0.001). With this subgroup, those with high GAS6 levels experienced all-cause death of 11.3% while those low GAS6 levels experienced mortality of 3.9%. Similarly, among patients with high NT-proBNP levels, mortality was 36.2% and 22.6% between GAS6 levels above and below the median, respectively. Therefore, circulating GAS6 levels may further distinguish the risks of adverse outcomes based on the NT-proBNP measurement. We found similar results when we use GAS6 and NT-proBNP to predict the prognostic information of cardiovascular death (Fig. 3; log-rank P < 0.001). Hs-TnT is another important biomarker of heart injury commonly used in clinical practice. We also found combination of GAS6 and hs-TnT could better differentiate patients at risk (Additional file 1: Figure S3).

### Analysis of renal dysfunction subgroups in patients with AHF

**Fig. 4:**
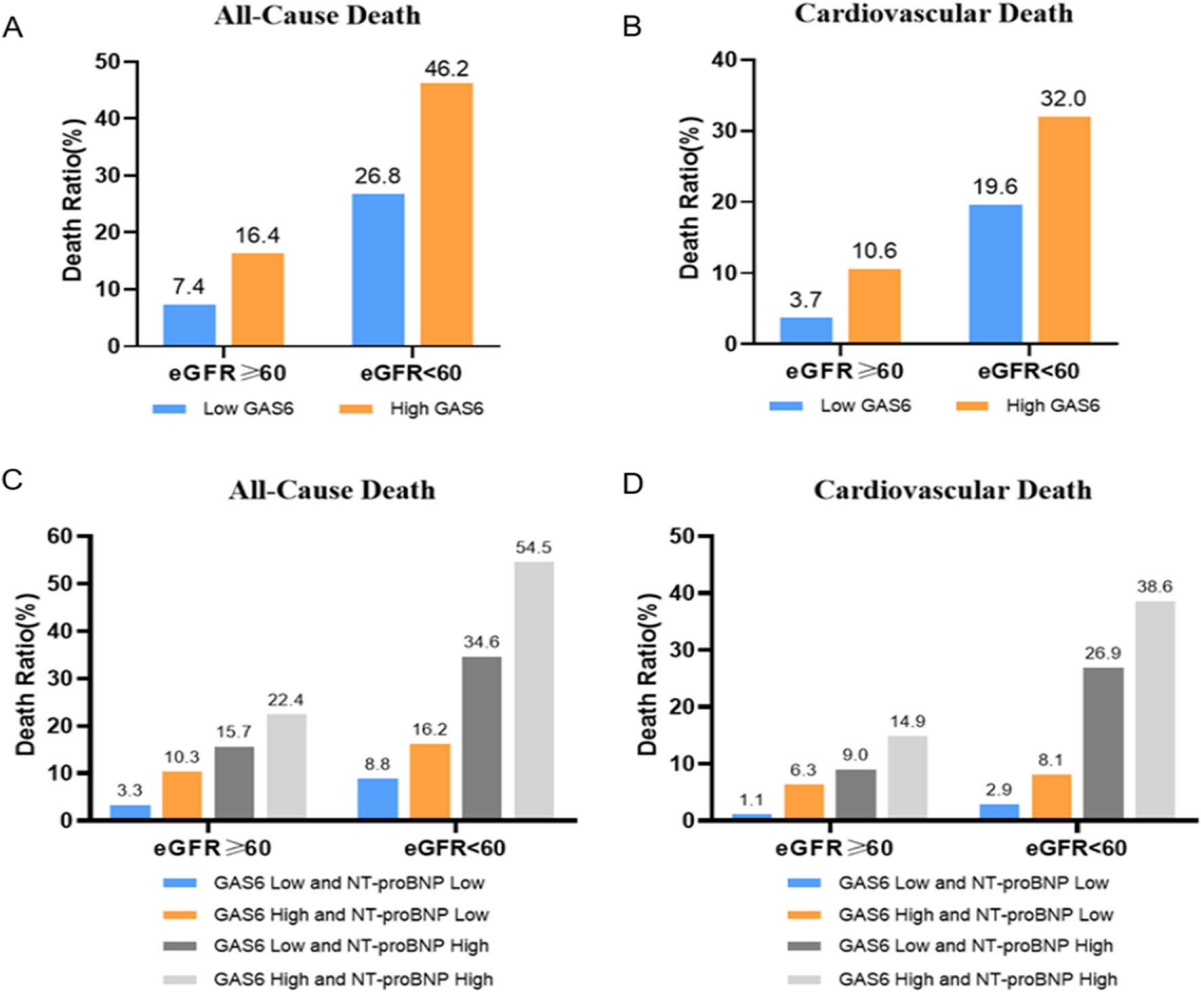
Observed incidence of all-cause death and cardiovascular death. Biomarker stratifications are as follows: the cut-off value of GAS6 level was 10.15 ng/mL, the cut-off value of NT-proBNP level was 1460.5 pg/mL. ‘High’ refers to GAS6/NT-proBNP levels above the cut-off value, while ‘low’ refers to GAS6/NT-proBNP levels below the cut-off value

**Fig. 5:**
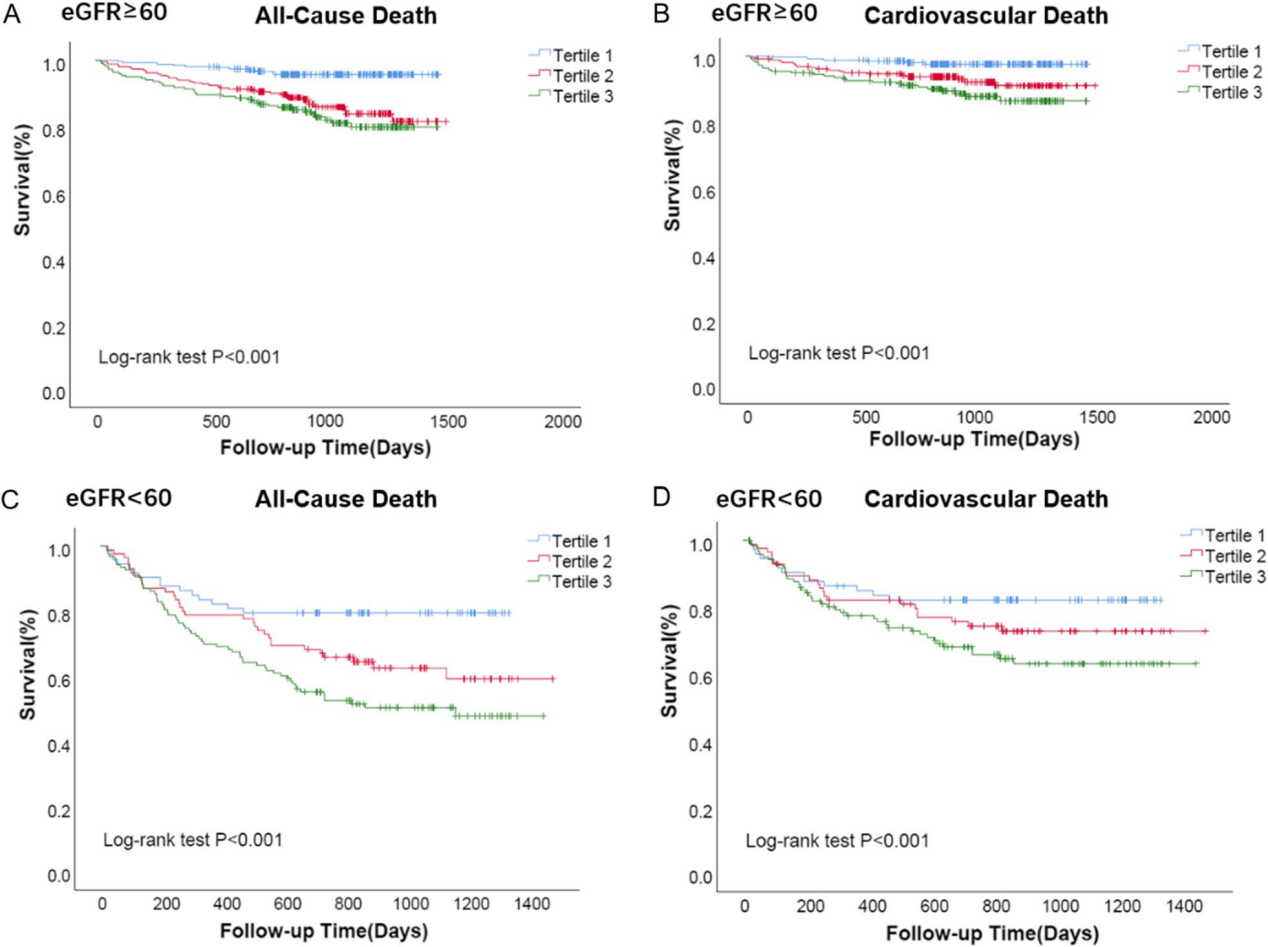
Survival regarding the primary endpoint (all-cause death) and the secondary endpoint (cardiovascular death) stratified according to the tertile distribution of GAS6 in different renal function groups (based on eGFR)

Previous study suggested that GAS6 was increased in CKD and kidney injury patients. We found GAS6 was negatively associated with eGFR (Additional file 1: Fig. S2). We classified AHF patients by history of renal dysfunction evaluated by eGFR < 60 mL/min/1.73m^2^. Characteristics analysis indicated that patients with renal dysfunction who died were older, and more likely to had worse cardiac and renal function assessed by LVEF and eGFR respectively. Furthermore, these patients who died had increased GAS6 levels, as well as BUN, hs-TnT, hs-CRP and NT-proBNP, with decreased BMI and hemoglobin in comparison to live patients (Table 4). High GAS6 or/and NT-proBNP levels were also associated with higher mortality (Fig. 4), the association was similar between GAS6 and other biomarkers, including BUN, hs-TnT, and hs-CRP (Additional file 1: Fig. S4). Survival analysis also revealed that higher GAS6 levels were more likely to be associated with adverse endpoints (Fig. 5; log-rank P < 0.001).

**Table 4:** Characteristics of patients with AHF classified by eGFR levels

	eGFR < 60			P value	eGFR ≥ 60			P value
	Death (n = 108)	Live (n = 173)	Total (n = 281)		Death (n = 85)	Live (n = 666)	Total (n = 751)	
GAS6, ng/mL	12.5 ± 3.6	10.4 ± 3.5	11.2 ± 3.7	< 0.001	11.8 ± 3.2	9.8 ± 3.4	10.0 ± 3.5	< 0.001
Age, years	77 ± 9	74 ± 12	75 ± 11	0.005	76 ± 11	69 ± 11	70 ± 12	< 0.001
BMI, kg/m^2^	23.6 ± 3.9	24.2 ± 4.0	24.0 ± 4.0	0.27	23.3 ± 3.5	24.6 ± 3.4	24.5 ± 3.0	0.002
HB, Hg/L	117 ± 22	122 ± 22	120 ± 22	0.07	126 ± 22	132 ± 16	131 ± 17	0.01
BUN, mmol/L	13.1 ± 5.4	10.7 ± 4.9	11.6 ± 5.0	< 0.001	7.3 ± 2.6	6.2 ± 1.8	6.3 ± 2.0	< 0.001
eGFR, mL/min/1.73m^2^	42 (27–51)	47 (37–54)	45 (35–53)	0.017	78 (66–93)	88 (74–104)	87 (73–102)	< 0.001
TnT, ng/mL	0.084 (0.043–0.420)	0.039 (0.023–0.082)	0.022 (0.013–0.059)	< 0.001	0.039 (0.019–0.261)	0.021 (0.013–0.057)	0.022 (0.013–0.059)	0.002
CRP, mg/L	7.8 (3.2–27.4)	5.0(3.0–17.1)	6.1(3.0–19.2)	0.053	3.5 (3.0–10.8)	3.2 (3.0–9.0)	3.2 (3.0–9.0)	0.806
NT-proBNP, pg/mL	7482 (3516–18,315)	2510 (912–5743)	3674 (1459–8946)	< 0.001	2436 (849–5182)	1083 (524–2272)	1163 (543–2527)	< 0.001
LVEF, %	40 ± 14	48 ± 14	45 ± 14	< 0.001	44 ± 13	48 ± 13	48 ± 13	0.006
LVMI, mm	129 ± 45	120 ± 37	123 ± 40	0.12	116 ± 42	111 ± 34	111 ± 35	0.286

GAS6: growth arrest specific 6; BMI: body mass index; Hb: hemoglobin; BUN: blood urea nitrogen; eGFR: estimate glomerular filtration rate; TnT: troponin T; CRP: C-reactive protein; NT-proBNP: N-terminal pro-B-type natriuretic peptide; LVEF: left ventricular ejection fraction; LVMI: left ventricular mass index. Death refers to all-cause death

## Discussion

Our study demonstrated for the first time that elevated plasma GAS6 levels were not only associated with impaired LV function, but also with higher all-cause and cardiovascular death in patients with AHF, which was independent of the established cardiac biomarker NT-proBNP and hs-TnT. Furthermore, we also revealed that AHF patients with renal dysfunction had higher plasma GAS6 levels.

GAS6 is a vitamin K-dependent protein and a kind of ligand of TAM family of tyrosine kinase receptors. As a decoy receptor for GAS6, increased sAXL levels undermine the effects of the GAS6 /AXL ligand interaction [22]. We previously demonstrated that the expression of GAS6 was upregulated in human dilated cardiomyopathy, and *Gas6* knockout in mice mitigated cardiac hypertrophy, fibrosis and contractile dysfunction. Conversely, cardiac-specific overexpression of *Gas6* aggravated the adverse phenotype [18]. Recent data suggest that GAS6 may play a pivotal role as prognostic biomarker in cardiac remodeling and LV function. According to a previous study, GAS6 levels were significantly lower during admission in STEMI patients compared with controls, and increased only from day 7, but there was no prognostic analysis of GAS6 and other risk factors [19]. Our study, to the best of our knowledge, is the first to assess the role of plasma GAS6 levels in prediction of adverse outcomes in patients with AHF. Plasma GAS6 in the acute phase in AHF patients, is closely associated with several risk factors of adverse prognosis in AHF patients, including NYHA functional class, hypertension and renal dysfunction (evaluated by eGFR). Therefore, GAS6 may represent an easily accessible biomarker of unfavorable prognosis in patients with AHF, and may provide further prognostic information based on the existing indicators.

Heart failure is a heterogeneous disease and also a common end-stage syndrome of different cardiovascular disorders. Although pressure and volume overloading as well as myocardial infarction constitute the most common causes of cardiac remodeling and heart failure, the myocardium manifest as compensatory enlargement in the initial phase of injury [23]. In this regard, GAS6 has been found to be crucial in mediating the deleterious effects following cardiac injury albeit expressed at varying levels in different disorders. During volume overloading, the change in GAS6 may not be pronounced following a mild inflammation /fibrosis and eccentric hypertrophic response. However, genetic ablation of GAS6 alleviated cardiac hypertrophy and improved cardiac function induced by pressure overloading [18]. A six months follow-up of STEMI patients revealed a potential role of the GAS6-AXL system in the pathophysiology of left ventricular remodeling following STEMI [19]. With regards to AHF, the ensuing neurohormonal, cytokine, inflammatory and oxidative stress storm ultimately leads to end-organ damage. Biomarkers of end-organ damage and dysfunction, such as troponins, cystatin-C and transaminases, have been shown to be increased in AHF and associated with poor prognosis [24]. And GAS6 has been demonstrated to be related to these biomarkers. GAS6 is mainly generated by cardiac and extracardiac tissues, while being correlated to activation of inflammatory processes, hemodynamic compromise, plaque instability, loss of vessel integrity, and tissue injuries, which significantly determine the progression of HF. Previous studies have showed exposure of human endothelial cells to 10% cyclical stretch increased release of the GAS6 and GAS6 /Axl signaling in human monocytes potentiated interleukin 1-beta production [25]. GAS6 could also increase the production of reactive oxygen species (ROS) in vascular smooth muscle cells, and mediate increased migration in vascular dysfunction [26]. Though not an organ-specific protein, the association between GAS6 and myocardial injury indicates that GAS6 may potentially function as a new and additional indicator of injury severity in AHF patients.

By further classifying GAS6 and NT-proBNP with median levels, the prognostic risk-stratification of adverse outcomes may be further enhanced. As natriuretic peptide release is mainly stimulated by pressure and /or volume overload, this will imply that GAS6 may be involved in other pathophysiological pathways regulating myocardial adaptation and dysfunction, as opposed to solely relying on the BNP-related pathway. GAS6 may thus provide more clinical information in patients with AHF independent of pressure or volume overload. Although GAS6 levels were unlikely influenced by infectious inflammatory processes in our study, we also cannot preclude the possibility that GAS6 levels might be influenced by other unknown processes in our cohort patients.

AHF is often accompanied by renal insufficiency, which suggests a worse prognosis in AHF patients. However, the predictive value of plasma biomarkers in adverse cardiovascular events and renal dysfunction is poor. Biomarkers, such as blood urea nitrogen, creatinine and uric acid, cannot provide accurate information about renal injury. Patients with renal dysfunction usually deteriorate rapidly due to improper diagnosis and treatment. Due to limitations of our study, the etiology in patients with renal dysfunction in our cohort was not easily identified. Nevertheless, GAS6 was found to be negatively associated with eGFR, and significantly elevated in AHF patients with renal dysfunction, especially in dead patients. Survival analysis also revealed that higher levels of GAS6 were associated with lower survival rates. We also found that higher levels of GAS6 and clinical biomarkers, including NT-proBNP, TnT, BUN and CRP, were accompanied by highest mortality in AHF patients, especially in those with renal dysfunction. In addition, GAS6 plays a pivotal role in pulmonary, hepatic and renal inflammation and fibrosis [27–29]. In this regard, AHF patients with renal dysfunction whose circulating GAS6 levels may indicate a primary inflammatory/fibrotic susceptibility may benefit from optimization of anti-inflammatory/antifibrosis therapies, such as mineralcorticoid receptor antagonists.

## Limitations

Our analysis has several limitations. First, there was no significant correlation between GAS6 stratification and HF re-hospitalization, which may lead to undue HF readmission. Second, there is limited information available on patient co-morbidities (especially chronic inflammatory conditions), which may potentially influence GAS6 levels. Third, serial measurement of GAS6 may provide more information about the association between GAS6 levels and prognosis of AHF patients. Fourth, we did not examine the effects of medical intervention on prognosis of GAS6. Anti-coagulation drugs, such as warfarin may influence GAS6 activation and levels. Fifth, we did not monitor continuous changes of eGFR nor determine the etiology of renal dysfunction.

## Conclusion

GAS6 may prove to be a novel and additional clinical biomarker for risk stratification of AHF patients, especially in the cohort with renal dysfunction. A combination of GAS6 and other clinical biomarkers might further pave the path forward to formulate precision interventions targeting the different pathways. It may be worthwhile exploring the accuracy and efficiency of diagnosis and prognosis of GAS6 specifically in patients with other cardiovascular diseases in the future.

## Supplementary Information

**Additional file 1: Table S1.** Univariate Cox regression between variables and clinical outcomes. **Table S2.** Multivariate Cox regression between variables and clinical outcomes. **Table S3.** Multivariate Cox regression between variables and clinical outcomes in patients with or without renal dysfunction. **Figure S1.** Hazard Ratios for All-Cause Death or Cardiovascular death According to GAS6 Deciles. **Figure S2.** Correlations between GAS6 and eGFR levels in the whole population indicated by scatter plot. **Figure S3.** Survival of the primary endpoint (all-cause death) and the secondary endpoint (cardiovascular death) stratified by median baseline GAS6 and hs-TnT levels. Kaplan–Meier analysis stratified by median baseline GAS6 and hs-TnT levels. Baseline median GAS6 was 10.15 ng/mL; the median level for hs-TnT was 0.029 ng/mL. ‘High’ refers to values above the median, while ‘low’ refers to values below the median. **Figure S4.** Observed incidence of all-cause death and cardiovascular death. Biomarker stratifications are as follows: the cut-off value of GAS6 level was 10.15 ng/mL, the cut-off value of hs-TnT level was 0.029 ng/mL, the cut-off value of hs-CRP level was 3.47 mg/L, the cut-off value of BUN level was 6.70 mm/L.

## Data Availability

The datasets supporting the conclusions of this article are included within the article and its supplementary information files.

## Abbreviations

GAS6: Growth arrest specific 6
HF: Heart failure
AHF: Acute heart failure
TAM: Tyrosine kinase receptor family
STEMI: ST Elevation Myocardial Infarction
eGFR: Estimated glomerular filtration rate
LVEF: Left ventricular ejection fraction
BMI: Body mass index
ACEI: Angiotensin-converting enzyme inhibitors
ARB: Angiotensin receptor blocker
NT-proBNP: N-terminal pro-B-type natriuretic peptide
Hs-TnT: High-sensitive troponin T
Hs-CRP: High-sensitive C-reactive protein
BUN: Blood urea nitrogen
CKD: Chronic kidney disease
ROS: Reactive oxygen species
