# Supplementary material for: Plasma GAS6 predicts mortality risk in acute heart failure patients: insights from the DRAGON-HF trial
Source: J Transl Med. 2023 Jan 12;21:21. doi: 10.1186/s12967-022-03859-w (PMC9838057; doi:10.1186/s12967-022-03859-w)
Supplement: Supplementary file 1 — Additional file 1: Table S1. Univariate Cox regression between variables and clinical outcomes. Table S2. Multivariate Cox regression between variables and clinical outcomes. Table S3. Multivariate Cox regression between variables and clinical outcomes in patients with or without renal dysfunction. Figure S1. Hazard Ratios for All-Cause Death or Cardiovascular death According to GAS6 Deciles. Figure S2. Correlations between GAS6 and eGFR levels in the whole population indicated by scatter plot. Figure S3. Survival of the primary endpoint (all-cause death) and the secondary endpoint (cardiovascular death) stratified by median baseline GAS6 and hs-TnT levels. Kaplan–Meier analysis stratified by median baseline GAS6 and hs-TnT levels. Baseline median GAS6 was 10.15 ng/mL; the median level for hs-TnT was 0.029 ng/mL. ‘High’ refers to values above the median, while ‘low’ refers to values below the median. Figure S4. Observed incidence of all-cause death and cardiovascular death. Biomarker stratifications are as follows: the cut-off value of GAS6 level was 10.15 ng/mL, the cut-off value of hs-TnT level was 0.029 ng/mL, the cut-off value of hs-CRP level was 3.47 mg/L, the cut-off value of BUN level was 6.70 mm/L. [file 12967_2022_3859_MOESM1_ESM.docx]

**Plasma GAS6 Predicts Mortality Risk in Acute Heart Failure Patients: Insights from the DRAGON-HF trial**

Teng Ma^1,2*^, Rongrong Huang^1*^, Yanhua Xu^1*^, Yangbo Lv^3^, Yifan Liu^1^, Xin Pan^4^, Jia Dong^5^, Di Gao^6^, Zeyu Wang^1^, Fenglei Zhang^2^, Chunxi Yan^2^, Sang-Bing Ong^7,8,9,10^, Yang Su^1^, Dachun Xu^1,2#^

**ADDITIONAL FILE**

**Table S1** Univariate Cox regression between variables and clinical outcomes.

|  | **95% CI** | **P value** |
| --- | --- | --- |
| **All-cause death** |  |  |
| **GAS6 (continuous)** | **1.18(1.13,1.22)** | **<0.001** |
| **GAS6 (categorical)** | **1.74(1.49,2.04)** | **<0.001** |
| NT-proBNP | 2.15(1.91,2.42) | <0.001 |
| TnT | 1.26(1.18,1.34) | <0.001 |
| CRP | 1.19(1.08,1.32) | <0.001 |
| Age | 1.05(1.04,1.07) | <0.001 |
| Sex | 0.85(0.63,1.14) | 0.27 |
| BMI | 0.93(0.89,0.97) | <0.001 |
| Smoking | 0.98(0.7,1.38) | 0.91 |
| Hypertension | 0.96(0.71,1.28) | 0.76 |
| Diabetes | 1.41(1.06,1.87) | 0.02 |
| Arterial Fibrillation | 0.68(0.48,0.96) | 0.03 |
| eGFR | 0.97(0.97,0.98) | <0.001 |
| Diuretics | 3.84(2.75,5.36) | <0.001 |
| Spironolactone | 2.49(1.85,3.34) | <0.001 |
| **Cardiovascular death** |  |  |
| **GAS6 (continuous)** | **1.19(1.13,1.24)** | **<0.001** |
| **GAS6 (categorical)** | **1.79(1.47, 2.17)** | **<0.001** |
| NT-proBNP | 2.36(2.04,2.73) | <0.001 |
| TnT | 1.29(1.19,1.39) | <0.001 |
| CRP | 1.22(1.08,1.39) | <0.001 |
| Age | 1.04(1.02,1.06) | <0.001 |
| Sex | 0.85(0.59,1.23) | 0.39 |
| BMI | 0.93(0.88,0.98) | 0.01 |
| Smoking | 1.01(0.67,1.53) | 0.97 |
| Hypertension | 0.93(0.65,1.34) | 0.7 |
| Diabetes | 1.69(1.2,2.4) | <0.001 |
| Arterial Fibrillation | 0.67(0.44,1.02) | 0.06 |
| eGFR | 0.97(0.96,0.98) | <0.001 |
| Diuretics | 5.17(3.29,8.11) | <0.001 |
| Spironolactone | 2.73(1.88,3.95) | <0.001 |

**Table S2** Multivariate Cox regression between variables and clinical outcomes.

|  | **95% CI** | **P value** | **c-index** |
| --- | --- | --- | --- |
| **All-cause death** | | | |
| Model1 | 1.15 (1.10,1.20) | <0.001 | 0.70(0.66,0.74) |
| Model2 | 1.13 (1.08,1.19) | <0.001 | 0.79(0.76,0.82) |
| Model3 | 1.10(1.05,1.16) | <0.001 | 0.82(0.79,0.85) |
| **Cardiovascular death** | | | |
| Model1 | 1.17(1.11,1.23) | <0.001 | 0.68(0.64,0.73) |
| Model2 | 1.15 (1.08,1.21) | <0.001 | 0.81(0.77,0.85) |
| Model3 | 1.11 (1.04,1.18) | 0.001 | 0.84(0.81,0.87) |

Standardized hazard ratios, reflecting the instantaneous risk of the primary and secondary endpoint for continuous biomarkers levels.

Model 1: age, sex, GAS6;

Model 2: age, sex, BMI, smoking, hypertension, diabetes, atrial fibrillation, eGFR, diuretics, spirolactone, GAS6;

Model 3: age, sex, BMI, smoking, hypertension, diabetes, atrial fibrillation, eGFR, diuretics, spirolactone, GAS6, NT-proBNP, TnT, CRP.

**Table S3** Multivariate Cox regression between variables and clinical outcomes in patients with or without renal dysfunction.

**Patients with renal dysfunction** (eGFR<60 mL/min/1.73m^2^)

|  | **95% CI** | **P value** | **c-index** |  |  |
| --- | --- | --- | --- | --- | --- |
| **All-cause death** | | | |  |  |
| Model1 | 1.13 (1.07,1.19) | <0.001 | 0.62(0.57,0.67) |  |  |
| Model2 | 1.14 (1.08,1.22) | <0.001 | 0.71(0.65,0.76) |  |  |
| Model3 | 1.10 (1.03,1.17) | 0.003 | 0.77(0.72,0.82) |  |  |
| **Cardiovascular death** | | | |  | 0 |
| Model1 | 1.12 (1.05,1.19) | <0.001 | 0.61(0.54,0.67) |  |  |
| Model2 | 1.12 (1.04,1.21) | 0.004 | 0.71(0.65,0.77) |  |  |
| Model3 | 1.07 (0.99,1.16) | 0.105 | 0.80(0.75,0.86) |  |  |

**Patients without renal dysfunction** (eGFR≥60 mL/min/1.73m^2^)

|  | **95% CI** | **P value** | **c-index** |
| --- | --- | --- | --- |
| **All-cause death** | | | |
| Model1 | 1.16 (1.09,1.23) | <0.001 | 0.71(0.65,0.76) |
| Model2 | 1.13 (1.05,1.20) | <0.001 | 0.78(0.73,0.83) |
| Model3 | 1.11 (1.03,1.19) | 0.004 | 0.79(0.74,0.85) |
| **Cardiovascular death** | | | |
| Model1 | 1.19 (1.10,1.28) | <0.001 | 0.71(0.64,0.77) |
| Model2 | 1.16 (1.06,1.26) | 0.001 | 0.81(0.75,0.87) |
| Model3 | 1.14 (1.04,1.24) | 0.004 | 0.83(0.77,0.88) |

Standardized hazard ratios, reflecting the instantaneous risk of the primary and secondary endpoint for continuous biomarkers levels.

Model 1: age, sex, GAS6;

Model 2: age, sex, BMI, smoking, hypertension, diabetes, atrial fibrillation, eGFR, diuretics, spirolactone, GAS6;

Model 3: age, sex, BMI, smoking, hypertension, diabetes, atrial fibrillation, eGFR, diuretics, spirolactone, GAS6, NT-proBNP, TnT, CRP


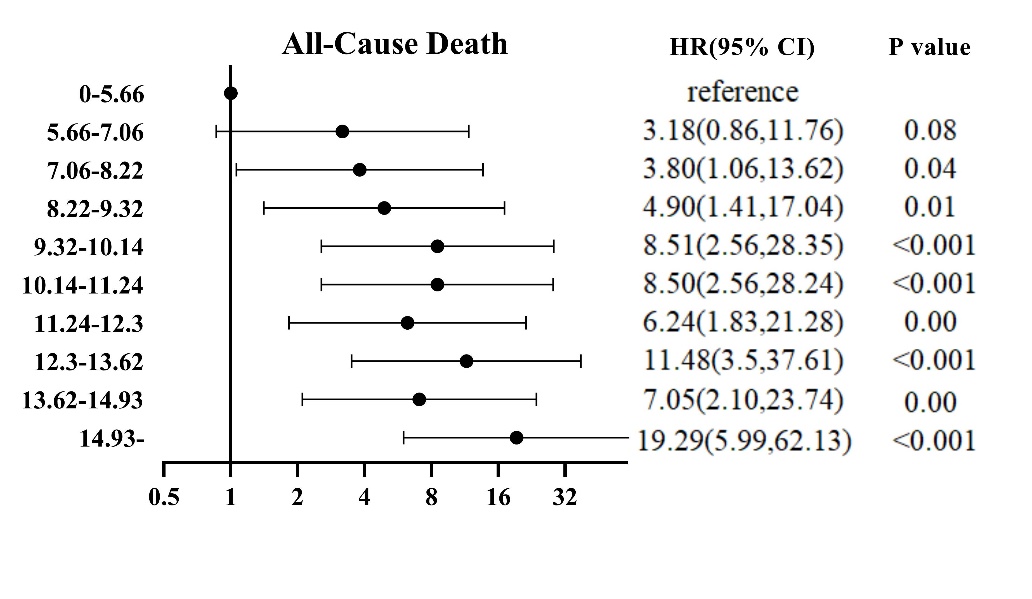
**Figure S1** Hazard Ratios for All-Cause Death or Cardiovascular death According to GAS6 Deciles


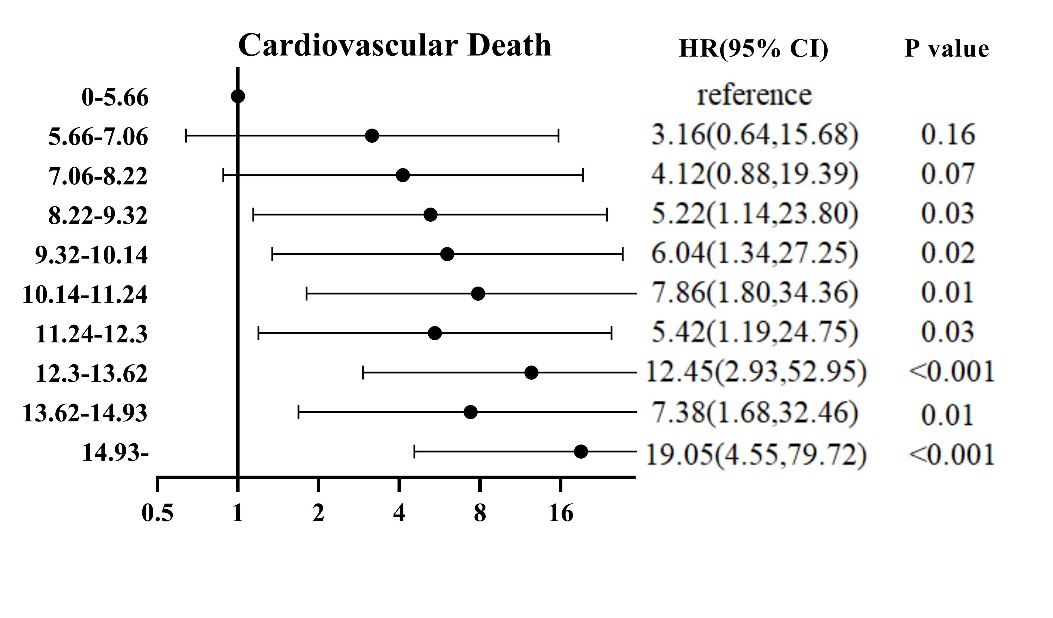
**(A)**

**(B)**


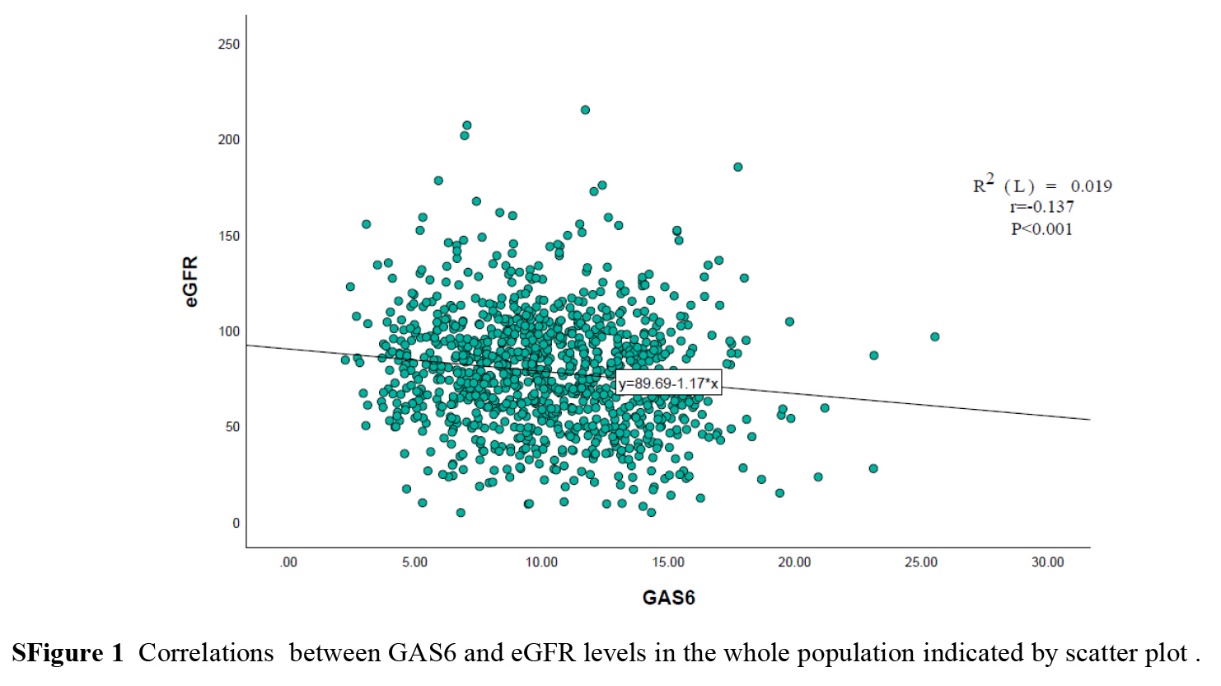
**Figure S2:** Correlations between GAS6 and eGFR levels in the whole population indicated by scatter plot.


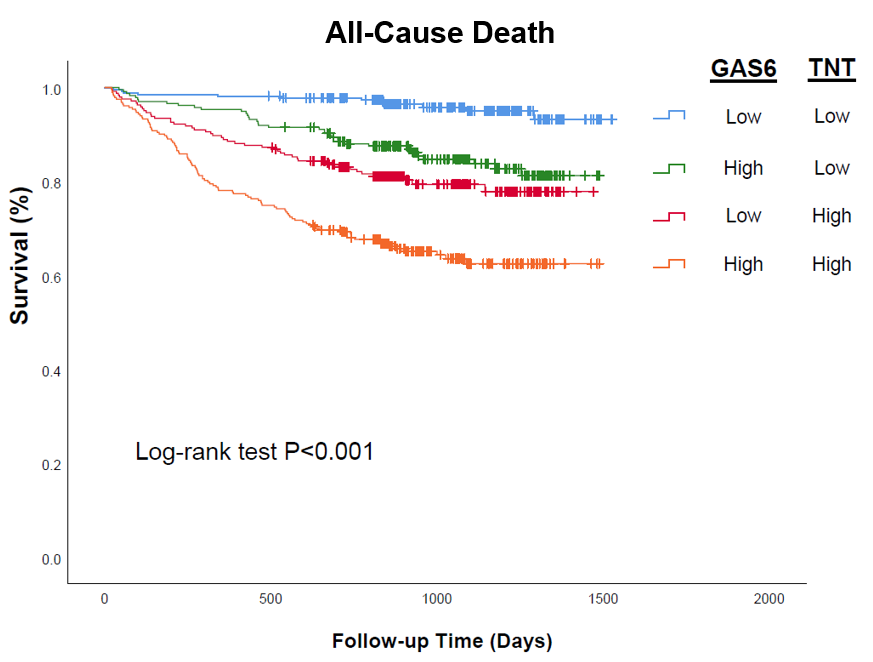
**Figure S3:** Survival of the primary endpoint (all-cause death) and the secondary endpoint (cardiovascular death) stratified by median baseline GAS6 and hs-TnT levels. Kaplan–Meier analysis stratified by median baseline GAS6 and hs-TnT levels. Baseline median GAS6 was 10.15 ng/mL; the median level for hs-TnT was 0.029 ng/mL. ‘High’ refers to values above the median, while ‘low’ refers to values below the median.

(A)


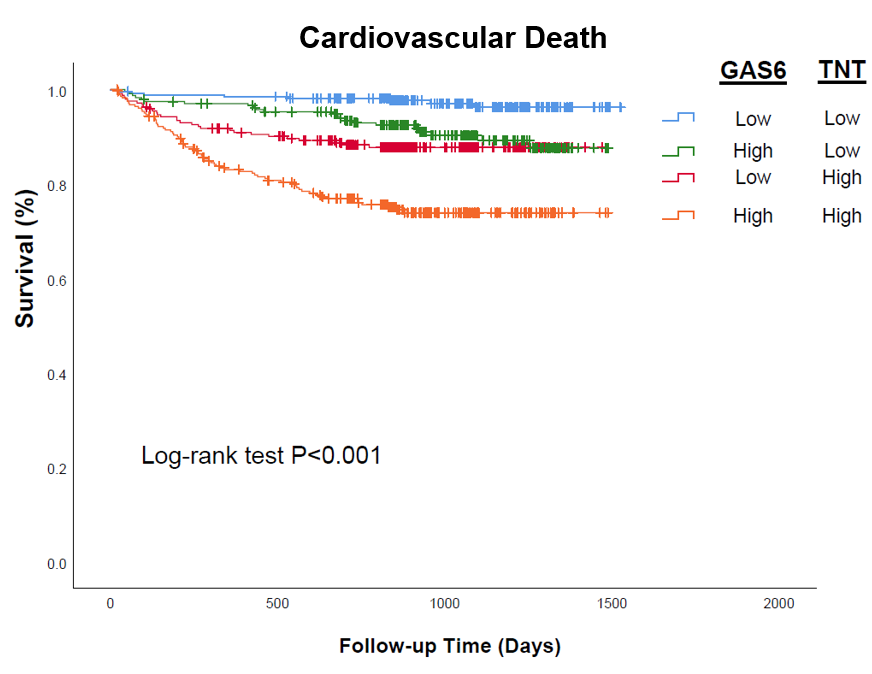


(B)

**
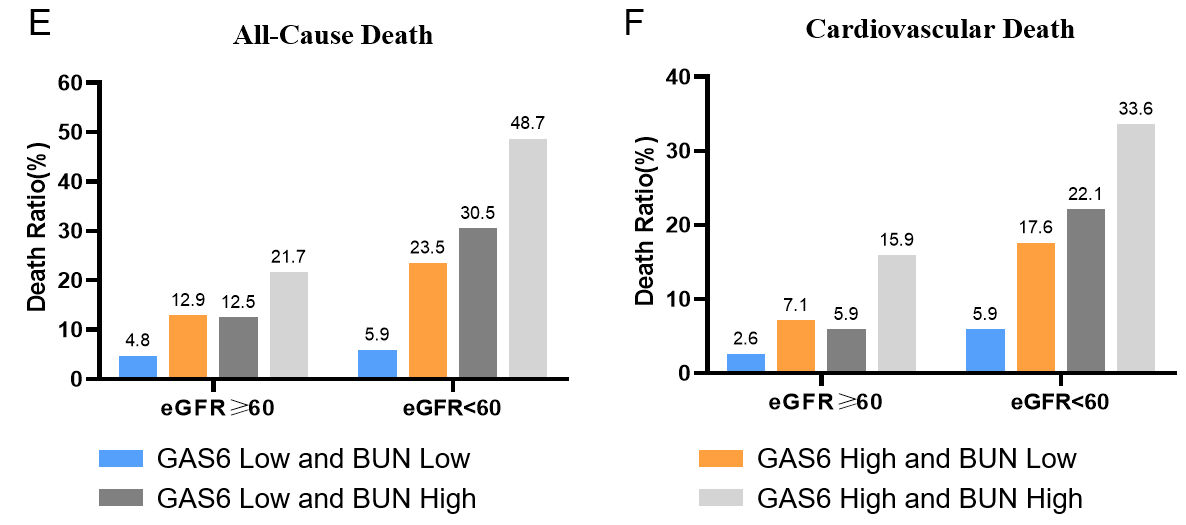
**
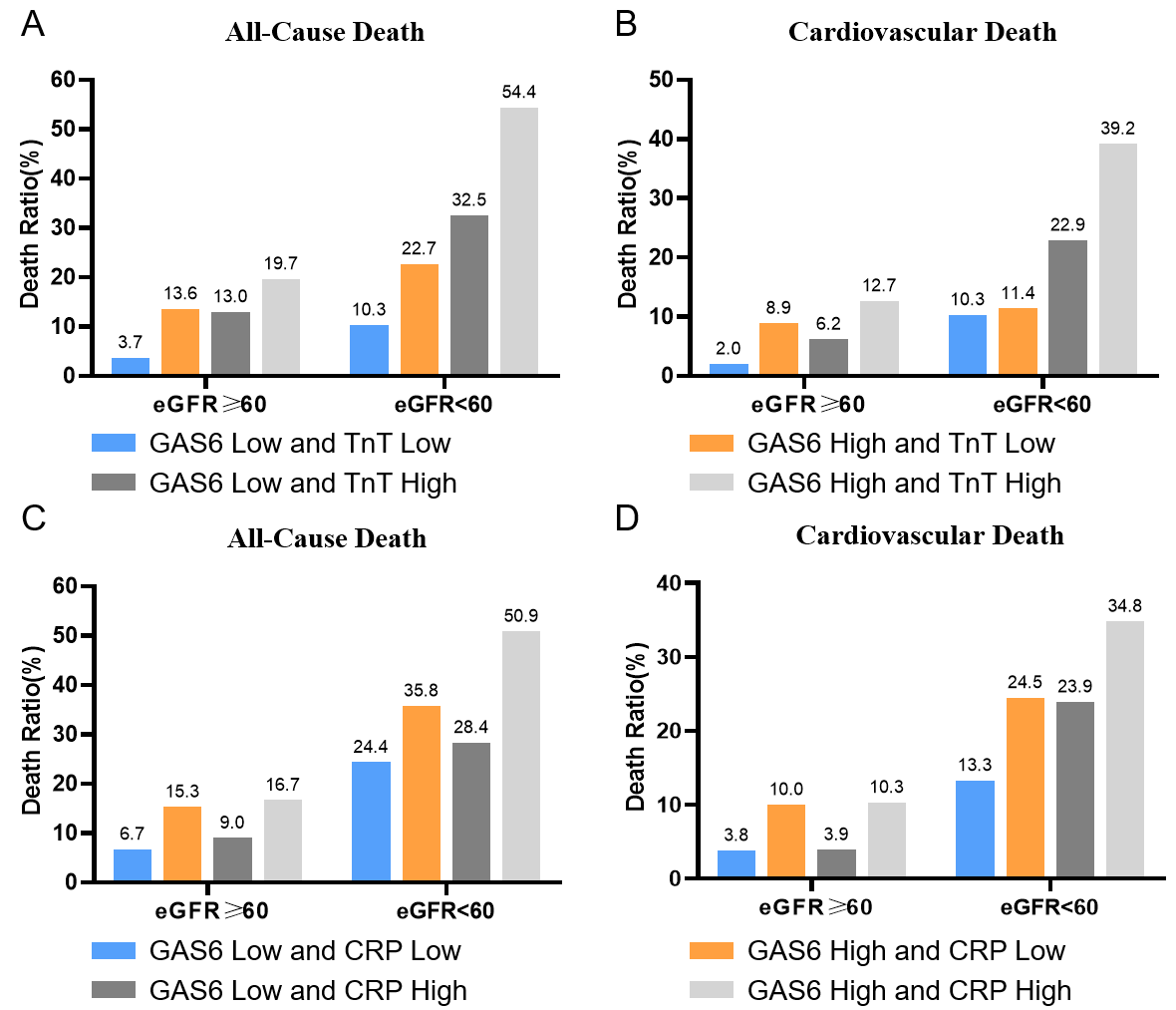
**Figure S4: Observed incidence of all-cause death and cardiovascular death. Biomarker stratifications are as follows: the cut-off value of GAS6 level was 10.15 ng/mL, the cut-off value of hs-TnT level was 0.029 ng/mL, the cut-off value of hs-CRP level was 3.47 mg/L, the cut-off value of BUN level was 6.70 mm/L.**
